# Type 2 diabetes in latin America: recommendations on the flash glucose monitoring system

**DOI:** 10.1186/s13098-024-01343-7

**Published:** 2024-05-20

**Authors:** Marcio Krakauer, Ana M. Gómez, Paloma Almeda-Valdes, Helard Manrique, María Lidia Ruiz Morosini, Gonzalo Godoy Jorquera, João Eduardo Nunes Salles, David Sanhueza Costa, Rodrigo de Azeredo Siqueira, Raquel N. Faradji, Alex Rincón Ramírez, Matías Ré, Karen Fériz Bonelo, Adrián Proietti, Fernando J. Lavalle-González

**Affiliations:** 1 Sociedade Brasileira de Diabetes, São Paulo, Brazil; 2 Pontifical Javeriana University, Bogotá, Colombia; 3 National Institute of Medical Sciences and Nutrition Salvador Zubirán, Mexico City, Mexico; 4 Delgado Clinic, Lima, Peru; 5 CODIME Center, Buenos Aires, Argentina; 6 University of Chile, Santiago, Chile; 7 Santa Casa de Sao Paulo School of Medical Science, Sao Paulo, Brazil; 8 Andrés Bello University, Santiago, Chile; 9 Rio de Janeiro Diabetes Center, Rio de Janeiro, Brazil; 10 ABC Medical Center, Mexico City, Mexico; 11 Pontifical Bolivarian University, Medellin, Colombia; 12 CINME Metabolic Research Center, Buenos Aires, Argentina; 13 Lili Valley Foundation, Cali, Colombia; 14 FASTA University, Mar del Plata, Argentina; 15 School of Medicine of the Autonomous University of Nuevo León, Nuevo León, Mexico

**Keywords:** Flash Continuous Glucose Monitoring System, Type 2 Diabetes, Consensus

## Abstract

**Objective:**

To establish recommendations through the consensus of a Latin American experts panel on the use of the flash glucose monitoring system (fCGM) in people living with type 2 diabetes mellitus (T2DM) regarding the benefits and challenges of using the fCGM.

**Methods:**

An executive committee of experts was created, comprised by a panel of fifteen physicians, including endocrinologists and internal medicine physicians, with expertise in management of adult patients with T2DM. The experts were from various countries: Colombia, Chile, Peru, Mexico, Argentina, and Brazil. The modified Delphi method was used, considering a consensus level of at least 80% of the participants. A seventeen-item instrument was developed to establish recommendations on the use of fCGM in patients with T2DM in Latin American.

**Results:**

The number of glucose scans recommended per day with the fCGM for patients managed with oral antidiabetic drugs or basal insulin was a median of 6 scans per day, and for those managed with multiple insulin doses, a median of 10 scans per day was recommended. Additionally, a holistic and individualized management approach was recommended, taking into account new treatment directions and identifying patients who would benefit from the use of the fCGM.

**Conclusion:**

Continuous use of the fCGM is recommended for people living with T2DM, regardless of their type of treatment. These metrics must be evaluated individually for each patient profile.

## Introduction

Type 2 diabetes mellitus (T2DM) is one of the major public health issues across Latin America, a region encompassing 21 countries and over 569 million inhabitants [1]. The International Diabetes Federation (IDF) reported that by 2021, approximately 341 million adults between the ages of 20 and 79 will live with diabetes in South and Central America, including the five countries with the highest number of persons living with diabetes being Brazil, Colombia, Venezuela, Argentina, and Chile. Moreover, Mexico has the second-largest adult population living with diabetes in North America and the Caribbean [2].

Glycemic control plays a crucial role in diabetes management, where glycosylated hemoglobin (HbA1c) remains the standard assessment tool, reflecting average blood glucose levels over an approximate span of three months [3]. However, HbA1c does not provide clinically relevant information, such as daily glucose variability or frequency of hyperglycemic and hypoglycemic episodes. HbA1c also lacks the specificity required to guide precise adjustments to oral antidiabetic drugs dosing [4].

These limitations can be overcome through fCGM, a practice gaining ground worldwide, supported by an expanding amount of evidence on its efficacy in T2DM management [5]. In addition to being a valuable tool for self-management of diabetes-encompassing facets like dietary management, physical activity, and dosage modulation-the reports generated by fCGM include important metrics such as time in range (TIR) and coefficient of variation (CV). These metrics provide a comprehensive view of glycemic control and glucose patterns, and in most studies, have shown a robust correlation with HbA1c [3].

Contrasting with traditional capillary blood glucose self-monitoring, which provides a singular blood glucose reading at a specific point in time, the fCGM employs factory-calibrated disposable sensors that continuously measures glucose levels at the interstitial fluid. Upon scanning the sensor with a reader or smartphone, users can access current glucose values and observe glucose patterns from the preceding 8 h, as well as trend arrows indicating the direction and velocity of blood glucose level fluctuations [6].

To make the most of fCGM devices, proper review and interpretation of data are essential. This requires active collaboration from both the person living with diabetes and the healthcare provider to ensure effective and timely utilization of the tracked data. However, there are still limitations regarding the availability of data and solid suggestions supporting the use of fCGM technology for T2DM management in Latin America [3].

We carried out a modified Delphi panel study to establish a consensus within Latin America on the fCGM in T2DM patients. The objective of the present work was to engage in discussions and gather insights on the advantages and challenges associated with using the fCGM. This initiative was driven by the absence of specific guidelines in Latin America for optimizing the use of the fCGM. Additionally, we aimed to derive recommendations on the profiles of patients who are most likely to benefit from the use of the fCGM.

## Methods

An Expert Executive Committee, comprised of a panel of fifteen doctors from Colombia, Chile, Peru, Mexico, Argentina, and Brazil, experts in T2DM management, was established. The main objectives were to review the existing literature and guidelines on fCGM utilization and develop a consensus document.

Initially, a systematic review of Clinical Practice Guidelines (CPG) from each included country was conducted, as well as international CPGs and guidelines addressing therapeutic goals for the management of patients living with T2DM. Afterwards, an instrument was developed to collect recommendations on monitoring procedures for T2DM management in Latin America [3, 7–15].

The instrument was divided into two sections. The first section presented 17 questions-both open-ended and closed-to gauge the experts perceptions using Likert scales ranging from: 1 (strongly disagree) to 5 (strongly agree). This section aimed to delve into areas such as current patient management, glycemic control objectives, and fCGM utilization. In the second section, participants engaged in creating a SWOT (Strengths, Weaknesses, Opportunities, and Threats) analysis for the fCGM in T2DM patients, employing brainstorming and rapid ideation grouping techniques.

Quality control of the content was conducted, followed by a descriptive statistical analysis. This analysis utilized measures of central tendency and dispersion to identify perception trends in the semi-quantitatively and qualitatively evaluated attributes, alongside dialogue analysis. For the calculation of consensus rates, each statement was treated equally, with no weighting applied. Consensus was established when 80% or more of the panelists rated a statement within either the agreement (4 and 5) or disagreement (1 to 3) thresholds. Following the first round, recommendations that did not meet the consensus criteria were discussed with the Consensus of Experts Group, proceeding to a second round of evaluation. Recommendations for which consensus could not be reached were declared as “no consensus,” with no additional evaluation undertaken.

## Results

The panel was primarily comprised of endocrinologists, representing 73% of the panel, while internal medicine physicians made up the remaining 27%. Regarding their professional practice locations, they were distributed as follows: 13% of the panelists in Chile, 7% in Peru, 20% in Colombia, 20% in Mexico, 20% in Argentina, and 20% in Brazil. In terms of gender, 67% of the panelists were male, while 33% were female. The session took place in August of 2023, and two rounds of anonymous interaction and open discussion were conducted for those recommendations that did not reach consensus in the first interaction. Recommendations for the use of the fCGM were divided in three main profiles: 1. Individuals living with T2DM managed with oral antidiabetic drugs; 2. Individuals living with T2DM managed with basal insulin; and 3. Individuals living with T2DM managed with multiple insulin doses.

### Current patient management and glycemic control goals

The initial discussion focused on capillary blood glucose measurement, a vital tool for self-monitoring and medication adjustment, especially crucial for patients administering insulin [3].

Recommendations (100% Consensus):

- Regarding the monitoring tools for glycemic control in adult patients with T2DM, it is recommended to: Consider metrics of glucose monitoring with individualized goals according to patient profile (life expectancy, comorbidities, economic resources), primarily taking into account 1. glycemic variability; 2. HbA1c, and 3. self-monitoring of capillary glucose.
- On the self-monitoring of capillary glucose, in individuals living with T2DM managed with oral antidiabetic drugs, at least one measurement per day is recommended. For those managed with basal insulin, a minimum of two measurements per day is recommended. In this population, it is essential to consider the available insulin treatments and differentiate the types available in each country, as this could modify the number of recommended measurements per day. For patients managed with multiple insulin doses, a minimum of 5 measurements per day is recommended.

### Use of the flash continuous glucose monitoring system

As fCGM becomes more widely adopted, it is necessary to standardize metrics and objectives to obtain consistent and effective reports of the results [16]. Specifically addressing the fCGM, the following recommendations were obtained from this consensus:

- The number of recommended daily glucose scans suggested for people living with T2DM undergoing treatment with oral antidiabetic drugs or basal insulin is a median of 6 (range: 5–14) scans per day, and for those on treatment with multiple insulin doses, a median of 10 (range: 6–20) scans per day is recommended (100% consensus).
- It is important to consider the recommendation of performing at least five readings: upon awakening, before each meal, and before going to bed, to avoid missing relevant information and to make effective joint decisions alongside patients. For patients who exercise, readings should also be performed before and after physical activity. Furthermore, it is crucial to educate all patients to understand that increasing the number of readings they perform, can improve their glycemic control metrics (100% consensus).
- In patients living with T2DM and managed with oral antidiabetic drugs, it is recommended to start the fCGM when the patient shows signs of poor glycemic control (100% consensus).
- For patients living with T2DM managed with basal insulin or multiple insulin doses, it is recommended to start the fCGM from the beginning of these treatments (100% consensus), when seeking to maintain optimal glycemic control (93% consensus), or when signs of macro or microvascular complications are detected (80% consensus).
- When considering changing a patient's therapeutic approach, physicians should evaluate their current treatment. For patients who are only managing their condition through diet and exercise, there is no consensus on the use of fCGM when switching to any other treatment. For patients who are managed with oral antidiabetic drugs (sulfonylureas), there are divergent opinions that prevent a consensus on specific recommendations regarding fCGM usage. This is mainly due to the fact that some countries have ceased prescribing sulfonylureas for T2DM management. Therefore, further evidence is required to demonstrate the benefits of using fCGM before making any definitive recommendations for this group of patients.
- Consider a holistic management of each patient and assess the direction of treatment changes and which patients actually require the use of the fCGM (100% consensus).
- Depending on the resources available at each country or if the patient maintains glucose levels within target range, the use of the fCGM for at least 14 days prior to consultation could be considered ideal. Additionally, it is important to highlight that, regardless of the type of ongoing treatment, the use of the fCGM should be maintained permanently to support therapeutic change strategies and lifestyle modifications (100% consensus).
- As for the recommended period for data review and interpretation obtained from the fCGM, the panel of experts has reached a consensus suggesting an interval ranging from every 14 days to every three months. The selected period will depend on the specific patient profile and taking into consideration their previous experience in using the sensor, management changes, and HbA1c levels (100% consensus).
- The ideal characteristics of a person living with T2DM to start the fCGM are summarized in Table 1.
- The aspects to consider when performing a review and interpretation of data from the fCGM are reported in Table 2.
- The main benefits of using the fCGM are shown in Table 3.
- Based on real-world experience and available evidence, Table 4 outlines the recommendations made by the Consensus of Experts for glycemic targets in all individuals living with T2DM.

**Table 1:** Ideal patient characteristics for fCGM monitoring initiation

Data review	Evidence level (consensus)
Basal insulin exclusively	High (100%)
Multiple insulin doses	High (93%)
Exclusively oral meditations (except sulfonylureas)	High (93%)
Frequent capillary monitoring in hospitalized patients	High (80%)
Concomitant diseases and high risk of hypoglycemia	High (100%)
Unnoticed, frequent, or severe hypoglycemia^†^	High (100%)
High-performance athletes	High (100%)
T2DM with HbA1c > 9%	High (93%)
T2DM and macrovascular complications	High (80%)
T2DM and microvascular complications	High (80%)

^†^For “Hypoglycemia unawareness”, consider if there is a suspicion that the patient may have presented hypoglycemia episodes that went unnoticed

**Table 2:** Recommended data evaluations in a patient using the fCGM

Data review	Evidence level (consensus)
Have data collected for at least 14 days	High (93%)
Time of use of fCGM over 70% based on days of sensor data captured; ideally 100%	High (100%)
Glycemic variability, determined by the variation coefficient (ideally < 36%) and standard deviation (ideally < 33%) of the average sensor value	High (93%)
Percentage of time within time in range (70–180 mg/dL) [ideally > 70%]	High (100%)
Percentage of time below range < 70 mg/dL (< 3.9 mmol/L) to less than 1 h per day, and time spent < 54 mg/dL (< 3.0 mmol/L) to less than 15 min per day	High (100%)
Percentage of time above range (> 180 mg/dL) ideally < 25%	High (100%)
Ambulatory glucose profile for hypoglycemia identification	High (100%)
Ambulatory glucose profile for hyperglycemia identification	High (100%)
Ambulatory glucose profile for glycemic variability identification	High (100%)
Daily profiles for pattern identification	High (100%)
Comparison of data from previous periods to evaluate changes	High (100%)

**Table 3:** Benefits of using fCGM

T2DM management	Clinical benefits	Benefits in healthcare sector	Level of evidence (consensus)
Oral antidiabetic drugs	• Lowering HbA1c • Time in range improvement • Educational tool • Lifestyle changes • Improved understanding of the condition • Improved treatment adherence	• Visualize the effects of food, exercise, and other factors • Early identification of glycemic excursions that warrant treatment intensification • Identification of hypoglycemia events when using antidiabetic drugs with this potential effect • Improved adherence • Patient education support	High (100%)
Basal insulin	• Lowering HbA1c • Time in range improvement • Delay progression of complications • Educational Tool • Lifestyle changes • Improved understanding of the condition • Improved treatment adherence	• Tracking across all metrics • Improved treatment adherence • Treatment titration, to avoid basal insulin overdose • Long-term control • Safety, by being able to visualize unnoticed hypoglycemic episodes and glucose fluctuations • Condition awareness	High (100%)
Multiple insulin doses	• Lowering HbA1c • Time in range improvement • Delay progression of complications • Educational tool • Lifestyle changes • Enhanced understanding of the condition • Improved treatment adherence • Support in identifying the need for carbohydrate intake adjustment	• Insulin dose adjustment • Prevention of hypoglycemia and fewer hospitalizations • Making appropriate adjustments (correction factor, insulin: carbohydrate ratio, basal dose adjustment) • Observing the effects of food and exercise • Long-term goals • Better control and fewer complications	High (100%)

**Table 4:** Glycemic goals with the fCGM

Data review	Evidence level (consensus)
Goals should be tailored based on the specific characteristics and settings of the device by country, as well as considering ethnic background, age, and other distinct/specific patient characteristics	High (87%)
Two metrics, time in range and time below range, should be used as starting points to assess the quality of glycemic control and as a basis for therapy adjustment. Emphasis should be placed on reducing time below range when percentage values drop below 54 mg/dL or 70 mg/dL, are near the targets, or exceed them	High (87%)
The use of the fCGM should be considered for all individuals with T2DM on intensive insulin therapy, whether it be through multiple daily injections or insulin pumps, taking into account patient-specific factors, preferences and resource availability	High (100%)
Continuous use of the fCGM, supplemented with confirmatory capillary blood glucose tests, is advised for people with T2DM who have high glycemic variability and are at high risk of hypoglycemia, including frequent, severe, or nocturnal events, or those who have hypoglycemia unawareness	High (93%)
The use of the fCGM can help lower HbA1c levels, reduce glycemic variability, and decrease episodes of hypoglycemia	High (100%)
The use of the fCGM can help educate and improve patient behaviors associated with dietary or lifestyle choices	High (100%)
The use of the fCGM can help optimize T2DM management	High (100%)
Compared to conventional glucose monitoring, the fCGM demonstrates superior effectiveness in glycemic control improvement, clinical outcomes, and quality of life for T2DM patients managed with basal insulin, with or without the addition of oral or injectable antidiabetic drugs	High (100%)
Continuous use of the fCGM may be beneficial in frail and/or older adults (age ≥ 65 years) with T2DM managed with basal insulin, with or without antidiabetic drugs (oral/injectable), as it can help detect hypoglycemia	High (100%)
The fCGM may be considered in pregnant women with T2DM who are on insulin therapy	High (93%)
For pregnant women with T2DM, the use of the fCGM starting from the first trimester until control goals are met, is recommended	High (100%)
Patients with T2DM experiencing poor glycemic control while on insulin therapy should consider using the fCGM, as this population is at a higher risk for hypoglycemia and may require significantly higher insulin doses	High (80%)
For all T2DM patients, the use of telemedicine and the fCGM is recommended to evaluate glycemic control when in-person consultation is not possible; this will allow the physician to understand glucose concentrations and their variations, and consequently, issue relevant instructions and recommendations	High (100%)

### SWOT analysis

**Fig. 1:**
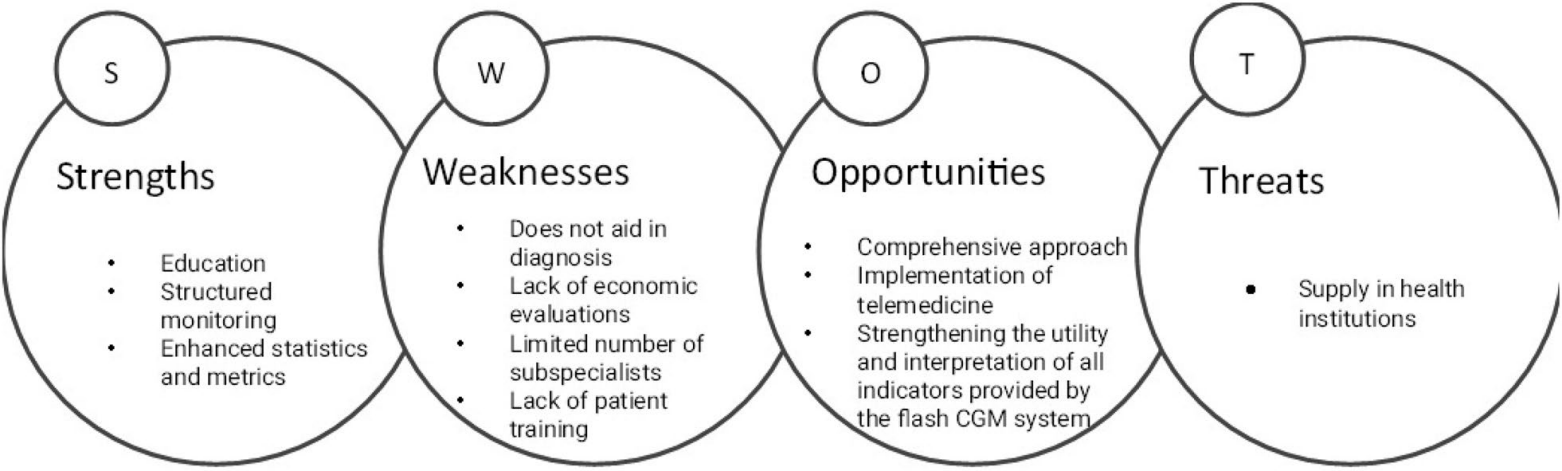
SWOT Analysis Results

In accordance with the analysis of the data obtained from the SWOT analysis, Fig. 1 reports the main perception considerations regarding the use of the fCGM system in people living with T2DM, according to expert opinions.

## Discussion

The recommendation on the use of the fCGM in patients living with T2DM has been established with a high degree of consensus. With regard to the number of daily glucose scans recommended for individuals with T2DM managed with non-insulin antidiabetic drugs or basal insulin, the panel suggests a range of 5 to 14 scans per day. For those managed with multiple insulin doses, the recommendation is a range of 6 to 20 daily scans. Furthermore, given that the typical duration of device use varied across Latin American countries, the recommendation is to consider using it for at least 14 days prior to consultation. The decision to extend its use permanently depends on the type of patient, treatment goals, and access and availability of the device.

The fCGM offers an innovative approach to glycemic management, yet their adoption and integration into clinical practice vary across Latin American countries. For instance, current recommendations in Mexico advise considering fCGM for individuals with type 2 diabetes who have inadequate glycemic control despite taking oral medications [27]. Similarly, the Brazilian Diabetes Society (SBD) recommends using fCGM as an additional tool for glycemic monitoring in the management of T2DM [15].

Two randomized, multicenter clinical studies comparing the combination of the fCGM plus education versus standard blood glucose self-monitoring were reported to show significantly greater decreases in HbA1c levels (adjusted risk difference -0.50%, 95% Confidence Interval (CI) − 0.74 to − 0.26, p < 0.001) and Time in Range (TIR) (2.4 h; 95% CI − 17.3% to − 2.5; p < 0.01) [17, 18].

In this context, the experts on the present report recommended training both physicians and patients regarding the use of the fCGM to take full advantage of the data provided by this system, performance of treatment adjustments to improve glucose levels, and enhance diet and quality of life. Comprehensive patient education and healthcare provider training are crucial for the successful implementation of fCGM in T2DM management.

Five studies evaluating the use of the fCGM in patients undergoing treatment with non-insulin antidiabetic agents or the combination with basal insulin reported statistically significant decreases in HbA1c levels compared to standard blood glucose self-monitoring. One of these studies also reported significant improvements in the Diabetes Self-Management Questionnaire (DSMQ) subscales, which include glucose management (p = 0.042), dietary control (p = 0.048), physical activity (p = 0.043), use of healthcare (p = 0.001), and self-care (p = 0.001), compared to baseline values [18–22].

In patients treated with multiple insulin doses, a randomized clinical trial reported reductions of 43% and 53% on the time spent in hypoglycemia < 3.9 mmol/L (70 mg/dL), reduced by 0.47 ± 0.13 h/day [mean ± SE (p = 0.0006)], and < 3.1 mmol/L (55 mg/dL), reduced by 0.22 ± 0.07 h/day (p = 0.0014), respectively [26]. Meanwhile, a prospective observational cohort study resulted in significantly reduced HbA1c after 3 to 6 months, with the use of the fCGM compared to the self-monitoring glucose group (0.3% ± 0.12 [3 mmol/mol ± 1.3], p = 0.0112) [5].

On the other hand, in six retrospective observational studies conducted in patients with basal insulin, significant decreases in HbA1c were also reported after implementing the fCGM [6, 23–25]. Additionally, one of the studies reported a decrease in the rates of acute diabetes-related events from 0.180 to 0.072 events/patient-year (Hazard ratio [HR]: 0.39 [0.30 to 0.51]; p < 0.001) and a reduction in hospitalization rates from 0.420 to 0.283 events/patient-year (HR: 0.68 [0.59 to 0.78]; p < 0.001) [6]. Another study also reported a 75% reduction in admissions for diabetic ketoacidosis and a 44% reduction in admissions for severe hypoglycemia [25] .

Based on the available evidence, the experts in the present report concluded that the fCGM system is a useful alternative to HbA1c for monitoring glycemic endpoints (TIR, hypoglycemia, hospitalizations, nutrition, exercise) in all patients living with T2DM compared to standard blood glucose self-monitoring.

## Conclusions

Based on this consensus exercise, the prescription of the fCGM is recommended for patients living with T2DM, taking into account all relevant metrics that can be evaluated with this type of monitoring. These metrics must be assessed on an individual basis, considering factors such as patient profiles and familiarity with the device. Whether the patient already has experience using the equipment or if they are a new patient, in which case training should be provided.

In summary, we can identify that the main strengths of using the fCGM are patient empowerment and an improved assessment of their condition through the various metrics the system can provide.

The integration of fCGM into T2DM management holds promise for improving glycemic control and reducing the risk of complications in Latin America. However, addressing challenges related to cost, accessibility, and education is essential to maximize its potential impact.

## Data Availability

Data is available at reasonable request to the corresponding author.

## Abbreviations

T2DM: Type 2 diabetes mellitus
IDF: International Diabetes Federation
HbA1c: Glycosylated hemoglobin
fCGM: Flash continuous glucose monitoring
TIR: Time in range
GMI: Glucose management indicator
CPG: Clinical Practice Guidelines
SGLT2i: Sodium-glucose cotransporter 2 inhibitor
SWOT: Strengths, weaknesses, opportunities, threats
DSMQ: Diabetes Self-Management Questionnaire
HR: Hazard ratio
